# Long-term analgesic use after neuroma and upper limb nerve injury: patterns in Sweden

**DOI:** 10.1097/PR9.0000000000001511

**Published:** 2026-10-28

**Authors:** Emma Dahlin, Raquel Perez, Malin Zimmerman, Juan Merlo, Erika Nyman, Lars B. Dahlin

**Affiliations:** a Department of Translational Medicine—Hand Surgery, Lund University, Skåne University Hospital, Malmö, Sweden; b Department of Orthopedics, Helsingborg Hospital, Helsingborg, Sweden; c Unit for Social Epidemiology, Department of Clinical Sciences (Malmö), Faculty of Medicine, Lund University, Malmö, Sweden; d Center for Primary Health Research, Region Skåne, Malmö, Sweden; e Department of Biomedical and Clinical Sciences, Linköping University, Linköping, Sweden; f Department of Orthopedic and Hand Surgery, Faculty of Medicine and Health, Örebro University, Örebro, Sweden; g Department of Hand Surgery, Skåne University Hospital, Malmö, Sweden

**Keywords:** Neuroma, Peripheral nerve injuries, Socioeconomic factors, Neuropathic pain, Analgesics, Opioid, Antidepressive agents, Tricyclic antidepressants, Serotonin and noradrenaline reuptake inhibitors, Surgical procedures

## Abstract

**Objectives:**

To examine demographic and socioeconomic differences in diagnosis and treatment of neuroma and nerve injury, assess risk of long-term use of centrally acting analgesics (CA-analgesics) compared with the general population, and describe how long-term use is distributed among individuals by demographic, socioeconomic, and treatment characteristics.

**Methods:**

In linked Swedish national registers (5,625,751 individuals, 25–80 years, residing 2010), incident cases of nonsurgically or surgically treated neuromas (n = 608‚ n = 189, respectively) or nerve injuries (n = 3,719, n = 2,122, respectively) were identified (no clinical data available) 2011 to 2014 and long-term CA-analgesic use was defined (prescription coverage ≥60% of days during 1 year; 60-day washout period after surgery). Multinomial and binary logistic regression models were used to estimate relative risks, adjusted for sex, age, income level, country of birth, and cohabiting. Absolute risks and absolute risk differences were calculated.

**Results:**

Neuroma or nerve injury cases were identified with variation in diagnosis and treatment by demographic and socioeconomic factors; women more likely to undergo neuroma surgery; men and younger individuals predominated among nerve injuries. All diagnostic treatment groups had markedly increased risks of long-term CA-analgesic use compared with the general population (adjusted relative risk range 3.5–6.0). Absolute risks among individuals exceeded 10% in socioeconomically disadvantaged subgroups (approximately 0.5% in the general population). Differences in long-term CA-analgesic use between nonsurgically and surgically treated individuals were modest.

**Conclusions:**

Neuroma and nerve injuries are associated with a substantial risk of long-term CA-analgesic use. Socioeconomic disadvantage strongly influences risk, whereas surgical treatment does not consistently reduce long-term medication use.

## Abstract 2

Supplemental Digital Content is Available in the Text.

Neuroma and nerve injuries are socially patterned with socioeconomically impacted risk of long-term use of centrally acting analgesics. Surgery does not consistently reduce such medication.

## 1. Introduction

Neuroma may develop after a traumatic nerve injury that is not adequately repaired, reconstructed, or treated^2,7^ or even if tethered–scarred by surrounding tissues.^17^ Nerve injuries and neuromas more commonly affect the upper limb, affecting hand function and quality of life.^10,27^ Some neuromas may lead to persistent pain.^8,50,52^ Neuropathic pain, which is a challenge to manage,^8,19,22,51^ contributes to considerable health care utilization and societal burden.^8,28^ Surgical intervention for neuroma may provide pain relief in selected individuals. However, symptoms and functional limitations often persist, which create uncertainty regarding long-term effectiveness.^5,10^ Nonsurgical treatment strategies may be less effective than surgery.^16^ Pharmacological management plays a central role and often includes the centrally acting analgesics (CA-analgesics), such as opioids, gabapentinoids (gabapentin, pregabalin), and selected adjuvant analgesics, including tricyclic antidepressants (TCAs, eg, amitriptyline), and serotonin–norepinephrine reuptake inhibitors (SNRIs; duloxetine and venlafaxine).^28^ According to Swedish national guidelines, amitriptyline, duloxetine, and venlafaxine are recommended for treating neuropathic pain. Opioids are discouraged because of limited long-term effectiveness and dependence risk^8^ and documented overuse in nerve entrapment disorders.^12,21^

Despite these recommendations, prescribing patterns for CA-analgesics among individuals with neuroma compared to traumatic nerve injuries and relative to the general population remain poorly characterized. It is unclear whether surgical treatment is associated with reduced long-term medication use, despite reports suggesting decreased opioid use after neuroma surgery in selected individuals.^26^ Demographic and socioeconomic factors may further influence both the risk of neuroma or nerve injury and subsequent treatment trajectories, including prolonged pharmacological pain management. Empirical evidence is limited.^5,18,34,36–38,43^

We hypothesised that demographic and socioeconomic characteristics are associated with risk of upper limb neuromas or nerve injuries, that these disorders are linked to an increased risk of long-term use of CA-analgesics, and that surgery may modify this risk. Accordingly, our aims were (1) to examine associations between demographic and socioeconomic factors and the likelihood of being diagnosed with an upper limb neuroma or nerve injury treated nonsurgically or surgically; (2) to assess risk of long-term use of CA-analgesics among affected individuals compared with the general population; and (3) to explore differences in long-term CA-analgesic use between nonsurgically and surgically treated individuals, while recognizing that treatment selection and unmeasured disease severity may confound this comparison.

## 2. Population and methods

### 2.1. Databases and population identification

Data were obtained through linkage of several Swedish national registers using a previously described and validated approach.^11,12^ These included the Total Swedish Population and the Longitudinal Integration Database for Health Insurance and Labour Market Studies, managed by Statistics Sweden (www.scb.se/en/), as well as the National Patient Register, the Cause of Death Register, and the Swedish Prescribed Drug Register, administered by the National Board of Health and Welfare (www.socialstyrelsen.se/en/). The linkage was approved by relevant data safety committees and authorized by the Regional Ethical Review Board in South Sweden (reference number 2014-856; data anonymized before access by researchers). The Swedish Prescribed Drug Register contains information about all drug dispensations in Swedish pharmacies (excluding stockpiles in nursing homes and hospital wards), coded according to the Anatomical Therapeutic Chemical (ATC) classification system. Meanwhile, the National Patient Register includes diagnoses from inpatient and specialized outpatient care coded using ICD-10 and records surgical and clinical procedures according to the Swedish version of the NOMESCO Classification of Surgical Procedures. The Total Swedish Population and the Longitudinal Integration Database for Health Insurance and Labour Market Studies database provide demographic and socioeconomic information.

All individuals, aged 25 to 80 years, residing in Sweden on December 31, 2010, were identified (n = 6,158,671; Fig. 1). We excluded individuals who died or emigrated before December 31, 2014, those who lacked information on country of birth, or had incomplete medication dosage data. Additional exclusions comprised individuals with mononeuropathies in the upper limb (ICD-10 G56), nerve tumours (ICD-10 D36.1, C47.1), a previous nerve injury, prior T-diagnoses related to upper limb nerve injury or amputation stump neuroma (ICD-10 T92.4, T87.3B–D), concurrent neuroma and nerve injury diagnoses, or evidence of long-term CA-analgesic use before baseline. The final study population included 5,625,751 individuals. During follow-up (2011–2014), 6,638 individuals were newly diagnosed with a nonsurgically or a surgically treated upper limb neuroma or nerve injury during 2011 to 2014 (Fig. 1; Table 1; Supplemental Table 1, https://links.lww.com/PR9/A447). This design allowed analyses both at the population level and within the subset of individuals with neuroma or nerve injury in line with study aims. Data on pain severity, symptom duration, indication for surgery, the precise characteristics of the neuroma, and functional impairment in the patients are not available in the registers.

**Figure 1.**
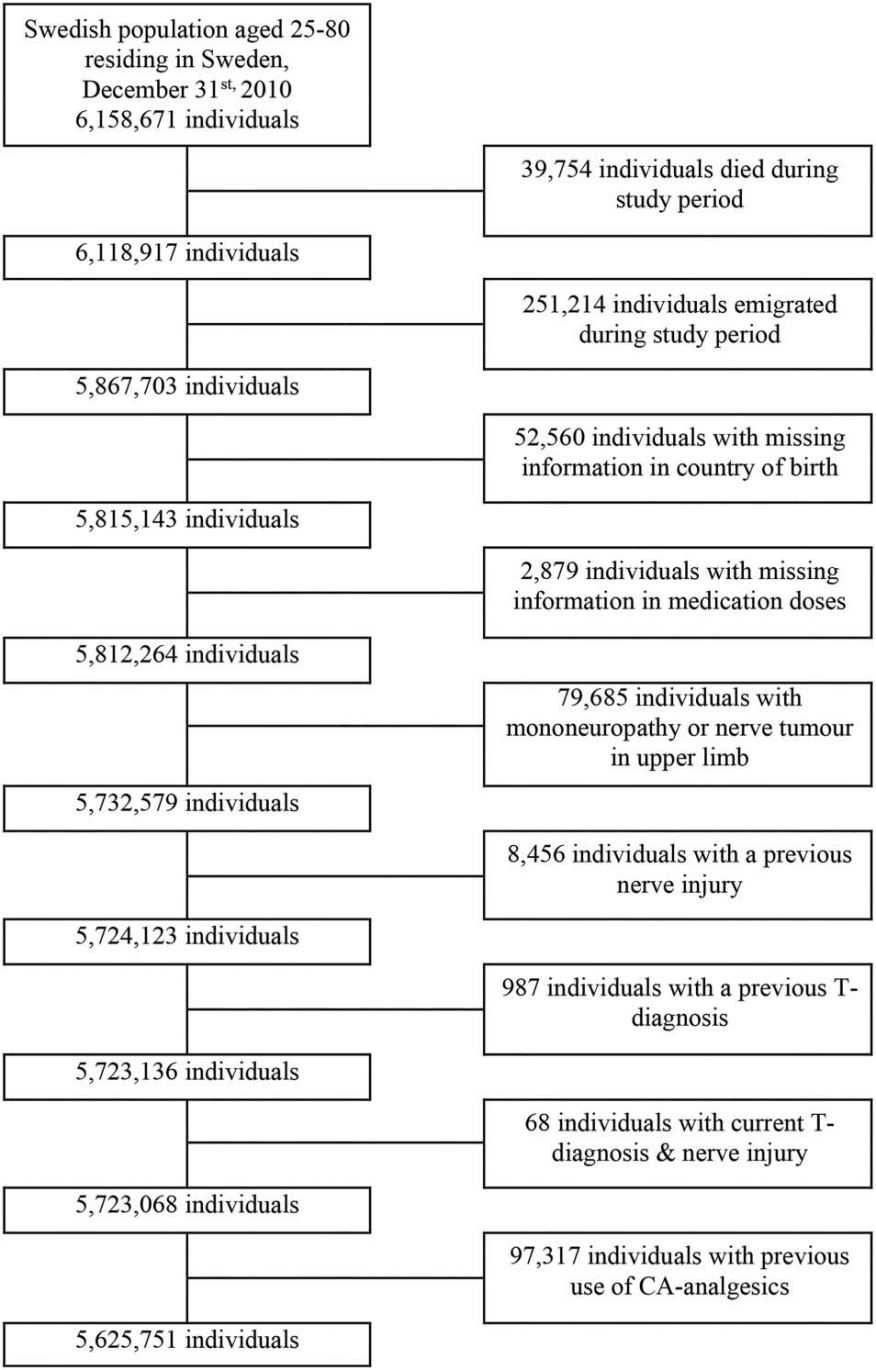
Flowchart showing the population that was analyzed concerning the likelihood for having a diagnosis of a neuroma or a nerve injury and a risk for consumption of centrally acting analgesics (CA-analgesics; ie, opioids, gabapentinoid drugs, and the 3 drugs amitriptyline, duloxetine, and venlafaxine used to treat neuropathic pain) in a Swedish population aged 25 to 80 years residing in Sweden, December 31, 2010 with focus on nonsurgically and surgically treated neuroma or nerve injuries in the upper limb.

**Table 1:** Demographic and socioeconomic characteristics of a population (with a diagnosis of nonsurgically or surgically treated neuroma or nerve injury in upper limb, as well as the existence of use of centrally acting–analgesics [CA-analgesics]) to treat neuropathic pain (ie, opioids, gabapentinoid drugs, amitriptyline, duloxetine, and venlafaxine) in a population aged 25 to 80 years, residing in Sweden by December 31, 2010 (n = 5,625,751 individuals).

Number		No neuroma or nerve injury (no surgery)	Nonsurgically treated neuroma	Surgically treated neuroma	Nonsurgically treated nerve injury	Surgically treated nerve injury
		5,619,113 (99.88)	608 (0.01)	189 (0.00)	3,719 (0.07)	2,122 (0.04)
Use of CA-analgesics	No high use	5,589,809 (99.48)	595 (97.86)	185 (97.88)	3,653 (98.23)	2,064 (97.27)
	High use	29,304 (0.52)	13 (2.14)	4 (2.12)	66 (1.77)	58 (2.73)
Sex	Men	2,823,369 (50.25)	346 (56.91)	75 (39.68)	2,299 (61.82)	1,516 (71.44)
	Women	2,795,744 (49.75)	262 (43.09)	114 (60.32)	1,420 (38.18)	606 (28.56)
Age (y)	25–34	1,002,824 (17.85)	99 (16.28)	34 (17.99)	681 (18.31)	526 (24.79)
	35–44	1,167,562 (20.78)	136 (22.37)	40 (21.16)	823 (22.13)	548 (25.82)
	45–54	1,131,599 (20.14)	124 (20.39)	52 (27.51)	814 (21.89)	475 (22.38)
	55–80	2,317,128 (41.24)	249 (40.95)	63 (33.33)	1,401 (37.67)	573 (27.00)
Income level	Low	1,293,315 (23.02)	149 (24.51)	54 (28.57)	922 (24.79)	538 (25.35)
	Middle–high	4,325,798 (76.98)	459 (75.49)	135 (71.43)	2,797 (75.21)	1,584 (74.65)
Country of birth (COB)	Swedish-born	4,820,550 (85.79)	501 (82.40)	159 (84.13)	3,235 (86.99)	1,826 (86.05)
	Immigrant	798,563 (14.21)	107 (17.60)	30 (15.87)	484 (13.01)	296 (13.95)
Cohabitation	Living alone	2,247,415 (40.48)	243 (39.97)	75 (39.68)	1,701 (45.74)	902 (42.51)
	Cohabiting	3,344,698 (59.52)	365 (60.03)	114 (60.32)	2,018 (54.26)	1,220 (57.49)

Values are numbers (percentages). For the definition of use, see Methods.

### 2.2. Assessment of variables

#### 2.2.1. Definition of nonsurgically or surgically treated neuroma and nerve injuries

In this study, the term *upper limb neuroma* was used as a register-based designation referring to clinically managed painful neuroma manifestations rather than histopathological entities. Specifically, “neuroma” was defined using 2 specific diagnoses and one additional internationally agreed neuroma definition for the surgically treated ones: (1) sequelae of nerve injury in the upper limb (ICD 10-code T92.4), (2) neuroma of the amputation stump in the upper limb (ICD 10-codes T87.3B, T87.3C, and T87.3D), and (3) the scarred–tethered nerve (included among the surgically treated neuroma; see below).^10,17^ For the latter, we included the subgroup of individuals with a diagnosis of traumatic nerve injury in the upper limb, excluding entrapped nerves, such as carpal tunnel syndrome or ulnar nerve entrapment (i.e., ICD codes G56), in whom surgical procedures involving nerve exploration or decompression were performed. These procedures were interpreted as a tethered or scarred nerve, a presentation that is often managed surgically in a manner analogous to neuroma treatment but may represent a more heterogeneous population than, e.g., an amputation neuroma. These tethered–scarred nerve (neuroma) were classified as a *surgically treated neuroma*^10,17^ (Supplemental Table 1, https://links.lww.com/PR9/A447).

To identify incident surgical episodes, we searched for nerve-related diagnoses and procedures occurring 5 years before the index surgery for everyone undergoing a first qualifying surgical procedure between January 1, 2011, and December 31, 2014. To ensure incident identification of nerve injury cases, individuals with nerve injury diagnoses recorded in the 5 years preceding the study period were excluded. Several surgical procedures were included in *surgically treated neuroma*, like nerve sutures, nerve reconstruction, nerve exploration, transection, and transposition, excision of nerve lesion, and nerve decompression (Supplemental Table 1, https://links.lww.com/PR9/A447). The procedures for nerve injuries included nerve sutures and reconstructions (Supplemental Table 1, https://links.lww.com/PR9/A447).

Individuals with similar diagnostic or procedural codes recorded in 2010 were excluded to ensure that only first-ever surgical episodes during the study period were included. The group with no neuroma or nerve injury and no nerve surgery served as the reference category in analyses of demographic and socioeconomic factors, likelihood of diagnosis, and long-term use of centrally acting analgesics. The temporal structure of episode identification and follow-up is illustrated in Figure 2.

**Figure 2.**
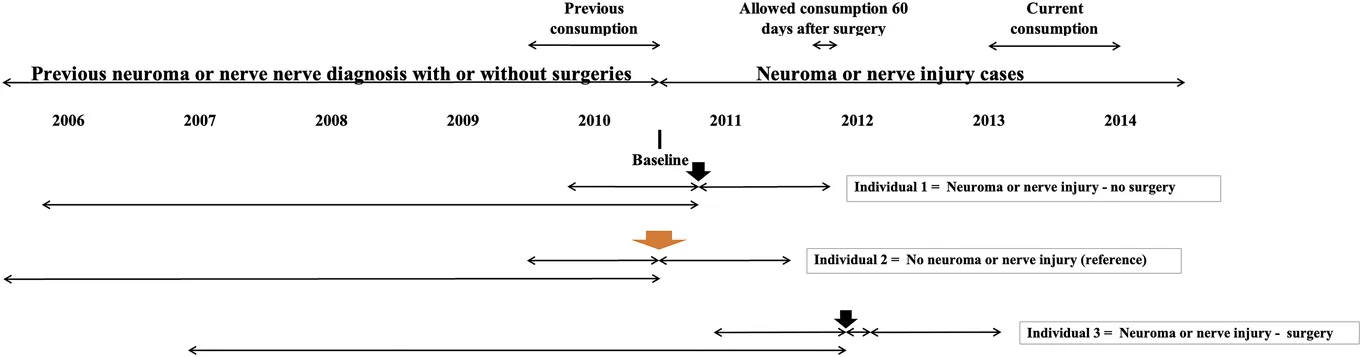
Schematic drawing of the principles of the use of centrally acting analgesics (CA-analgesics; ie, opioids, gabapentinoid drugs, amitriptyline, duloxetine, and venlafaxine) in individuals with nonsurgically and surgically treated neuromas or nerve injuries in the upper limb, with an explanation of how everyone was evaluated. The individuals without neuroma or nerve injury were sampled from December 31, 2010 (red arrow; individual 2), whereas the individuals with nonsurgically (individual 1) and surgically (individual 3) treated neuroma or nerve injury were sampled during 2011 to 2014, and each individual was followed for 1 year. For instance, the individual 1, *with a neuroma or nerve injury diagnosis but without surgery*, got the diagnosis on April 20, 2011, and then the follow-up for CA-analgesics use (i.e., the outcome) was from that point to April 20, 2012. In individual 1, we evaluated any previous consumption 1 year before from April 20, 2010 to April 20, 2011. For individual 2, *without any neuroma or nerve injury diagnosis (or surgery) between 2011 and 2014*, we assessed previous consumption during 2010, and then a follow-up for CA-analgesics use was observed in 2011. Individual 3, *with a neuroma or a nerve injury with surgery*, received their diagnosis on June 5, 2012, and any previous consumption was evaluated between June 5, 2011 and June 5, 2012, whereas the follow-up for CA-analgesics use was assessed from August 5 2012 to August 5, 2013 (i.e., 60 days consumption allowed after surgery).

#### 2.2.2. Demographic and socioeconomic factors

Demographic and socioeconomic characteristics were obtained from the linked national registers and included age, sex, country of birth, cohabitation status, and income. Age was categorized as 25 to 34 years (reference), 35 to 44 years, 45 to 54 years, and 55 to 80 years. Sex was classified as male (reference) or female. Country of birth was categorized as immigrant (reference) or Swedish-born. Cohabitation status was defined as cohabiting (married or living together at the same address; reference) or living alone.

Income was based on disposable family income and calculated as a cumulative measure using data from 2000, 2005, and 2010, accounting for household size and individual consumption weights according to Statistics Sweden methodology.^11,12^ Based on this measure, individuals were classified into low-, middle-, or high-income (reference) groups. Individuals with missing income data in 2000 or 2005 were assigned values from 2010; no individuals had missing income data for 2010.

For analyses of long-term CA-analgesic use using relative risk estimates, the income levels were categorized as high (reference), middle, or low. For analyses of absolute risks (ARs) and absolute risk differences (ARDs), income levels were dichotomized into low vs middle–high to improve interpretability and precision.

#### 2.2.3. Dispensation of pain medications—definition of centrally acting–analgesics

Dispensations of centrally acting–analgesics (CA-analgesics) were identified using ATC codes. Opioids were defined by the N02A group, which included morphine (N02AA01), oxycodone (N02AA05), oxycodone–naloxone (N02AA55), buprenorphine (N02AE01), ketobemidone and spasmolytics (N02AG02), fentanyl (N02AB03), opioid combinations with nonopioid analgesics (N02AJ), and codeine (R05DA04). In addition, gabapentin (N03AX12) and pregabalin (N03AX16; ATC coding according to study period) were comprised, as well as the antidepressants amitriptyline (N06AA09), duloxetine (N06AX21), and venlafaxine (N06AX16), which are commonly prescribed in Sweden for neuropathic pain. Accordingly, CA-analgesic use was defined as any dispensation of the above opioids, gabapentinoids, or antidepressants, either alone or in combination.

#### 2.2.4. Definition of long-term use of centrally acting–analgesics, washout period, and baseline analgesic use

Long-term use of centrally acting–analgesics (CA-analgesics) was defined as medication coverage exceeding 60% of days during a 1-year follow-up period; a previously used and valid definition in register-based pain medication studies.^12,14^ For each individual, coverage was estimated by dividing the cumulative number of dispensed defined daily doses by 365 days, and the outcome was coded as a binary indicator (yes/no).

To avoid misclassifying short-term postoperative prescribing as long-term use, a 60-day washout period was applied for surgically treated individuals. For these individuals, the index date was the date of surgery, and follow-up began 60 days thereafter.^14^ For nonsurgically treated individuals, the index date was the date of the first qualifying neuroma or nerve injury diagnosis, with follow-up beginning immediately. For individuals in the general population without a neuroma or nerve injury, the index date was December 31, 2010.

In all groups, the medication use was assessed over 1 year after the relevant index date (or after applied washout period for surgically treated individuals). Individuals with evidence of long-term CA-analgesic use before the index date were excluded to ensure that the outcome reflected incident long-term use during follow-up. Specifically, prior CA-analgesic use was assessed during the 1 year preceding the index date for all individuals. Those whose medication coverage exceeded 60% of days during this 1-year lookback period were excluded from the analysis.

### 2.3. Statistical analyses

#### 2.3.1. Descriptive analyses

First, we described the study population by presenting number and percentage of individuals in each diagnostic–treatment category (nonsurgically treated neuroma, surgically treated neuroma, nonsurgically treated nerve injury, surgically treated nerve injury, and the general population without neuroma or nerve injury; for definition of the 3 types of neuroma, see above), together with demographic and socioeconomic characteristics and the proportion with long-term CA-analgesic use (Table 1).

#### 2.3.2. Aim (a): likelihood of diagnosis and treatment

Second, associations between demographic and socioeconomic factors and likelihood of being diagnosed with an upper limb neuroma or nerve injury, and of being treated nonsurgically or surgically, were examined using multinomial logistic regression. The 4 diagnostic–treatment categories were compared simultaneously with the general population (reference). Models included sex, age, income level, country of birth, and cohabitation status, allowing estimation of independent associations for each factor. Results are presented as odds ratios (ORs; presented as relative risks [RRs], see below for explanations) with 95% confidence intervals (CIs) (Table 2).

**Table 2:** Relative risks associated with demographic and socioeconomic factors of the likelihood of having a diagnosis of nonsurgically or surgically treated neuroma or nerve injury in the upper limb in a Swedish population (25–80 years, residing in Sweden by December 31, 2010; n = 5,625,751 individuals).

		Nonsurgically treated neuroma (n = 608)	Surgically treated neuroma (n = 189)	Nonsurgically treated nerve injury (n = 3,719)	Surgically treated nerve injury (n = 2,122)
Sex	Men	Reference	Reference	Reference	Reference
	Women	0.76 (0.64–0.89)	1.51 (1.13–2.03)	0.64 (0.60–0.68)	0.42 (0.38–0.46)
Age (y)	25–34	Reference	Reference	Reference	Reference
	35–44	1.19 (0.92–1.55)	1.04 (0.65–1.66)	1.12 (1.01–1.24)	0.91 (0.81–1.03)
	45–54	1.12 (0.85–1.46)	1.38 (0.89–2.14)	1.11 (1.00–1.22)	0.81 (0.72–0.92)
	55–64	1.23 (0.94–1.61)	1.14 (0.71–1.83)	1.03 (0.93–1.15)	0.63 (0.55–0.72)
	65–80	1.03 (0.79–1.36)	0.61 (0.36–1.04)	0.89 (0.80–0.99)	0.38 (0.31–0.43)
Income	High	Reference	Reference	Reference	Reference
	Middle	1.09 (0.91–1.32)	1.31 (0.93–1.85)	1.14 (1.05–1.24)	1.17 (1.05–1.31)
	Low	1.11 (0.89–1.38)	1.43 (0.97–2.09)	1.00 (0.93–1.08)	1.04 (0.94–1.15)
Country of birth (COB)	Immigrant	Reference	Reference	Reference	Reference
	Swedish-born	0.79 (0.64–0.98)	0.98 (0.66–1.47)	1.14 (1.04–1.26)	1.08 (0.95–1.22)
Cohabiting	Yes	Reference	Reference	Reference	Reference
	No	1.00 (0.85–1.18)	1.02 (0.76–1.37)	1.27 (1.19–1.35)	1.03 (0.94–1.12)

The relative risk (RR) and 95% confidence intervals (CI) are presented.

#### 2.3.3. Aim (b): long-term centrally acting–analgesic use in the population

Third, the risk of long-term CA-analgesic use among individuals with neuroma or nerve injury was assessed using binary logistic regression, with long-term use (yes/no) during 1 year of follow-up as the outcome and diagnostic–treatment group as the exposure (reference: general population). We present results from an unadjusted model (Model 1) and a model including sex, age, income level, country of birth, and cohabitation status (Model 2) to assess whether differences persisted after accounting for demographic and socioeconomic composition (Table 3).

**Table 3:** Relative risks of long-term use of centrally acting–analgesics (CA-analgesics; ie, opioids, gabapentinoid drugs, amitriptyline, duloxetine, and venlafaxine used to treat neuropathic pain) in nonsurgically or surgically treated neuroma or nerve injury in upper limb in a Swedish population (25–80 years, residing in Sweden by December 31, 2010; n = 5,625,751 individuals).

		Model 1 (unadjusted)	Model 2 (adjusted)
Cases	No neuroma or nerve injury (no nerve surgery)	Reference	Reference
	Nonsurgically treated neuroma	4.17 (2.41–7.22)	4.24 (2.45–7.35)
	Surgically treated neuroma	4.12 (1.53–11.11)	3.89 (1.44–10.50)
	Nonsurgically treated nerve injury	3.47 (2.73–4.42)	3.52 (2.76–4.49)
	Surgically treated nerve injury	5.34 (4.10–6.95)	6.01 (4.63–7.81)
Sex	Men		Reference
	Women		1.42 (1.39–1.45)
Age (y)	25–34		Reference
	35–44		1.46 (1.40–1.52)
	45–54		1.62 (1.56–1.69)
	55–64		1.83 (1.76–1.91)
	65–80		1.78 (1.71–1.85)
Income	Low		1.98 (1.92–2.04)
	Middle		1.49 (1.45–1.53)
	High		Reference
Country of birth	Immigrant		Reference
	Swedish-born		1.07 (1.03–1.10)
Cohabiting	Yes		Reference
	No		1.49 (1.46–1.53)
AUC value		0.502 (0.501–0.502)	0.619 (0.616–0.622)

The relative risk (RR) and 95% confidence intervals (CI) are presented as unadjusted (Model 1) and adjusted (Model 2) values.

AUC, area under the receiver operating characteristic curve.

Because both diagnoses and long-term CA-analgesic use were rare (<3%), ORs were interpreted as approximations of RRs and are presented as RRs with 95% CIs.

#### 2.3.4. Aim (c): distribution of long-term centrally acting–analgesic use within individuals and by treatment status

Fourth, among individuals with neuroma or nerve injury, we estimated ARs and ARDs of long-term CA-analgesic use. Analyses were stratified by demographic and socioeconomic factors and by nonsurgical vs surgical treatment status, using stratified logistic regression. For these analyses, income was categorized as low vs middle–high, and age as 25 to 34, 35 to 44, 45 to 54, and 55 to 80 years (Table 4). Comparisons by surgical status were interpreted descriptively, given the observational design and potential treatment selection.

**Table 4:** Distributions of centrally acting analgesics in individuals with surgically untreated and treated neuroma or nerve injury.

Prescribed drugs	Detailed names of drugs	ATC-codes	Numbers (%)*	Distribution (%)†
Opioids	Morphine	N02AA01	100,069 (10.34)	67
	Oxycodone	N02AA05	215,921 (22.31)	
	Oxycodone and naloxone	N02AA55	6,470 (0.67)	
	Buprenorphine	N02AE01	60,825 (6.28)	
	Ketobemidone and spasmolytics	N02AG02	1,832 (0.19)	
	Fentanyl	N02AB03	20,083 (2.07)	
	Opioids in combination with nonopioid analgesics	N02AJ	175 (0.02)	
	Other opioids (tramadol and tapentadol)	N02AX	554,797 (57.32)	
	Codeine	R05DA04	7,793 (0.81)	
Gabapentinoids	Pregabalin	N03AX16	119,819 (62.66)	13
	Gabapentin	N03AX12	71,412 (37.34)	
Tricyclic antidepressant	Amitriptyline	N06AA09	142,330 (50.27)	19
Serotonin–norepinephrine reuptake inhibitors (SNRI)	Venlafaxine	N06AX16	84,351 (29.79)	
	Duloxetine	N06AX21	56,468 (19.94)	

* Percentage relates to distribution within the different categories.

† Percentage relates to the distribution between different categories.

ATC, Anatomical Therapeutic Chemical.

### 2.4. Model discrimination

Finally, model discrimination for long-term CA-analgesic use was assessed using the area under the receiver operating characteristic curve (AUC), with conventional thresholds used to interpret discriminatory ability (AUC values: 0.50–0.60 absent or very weak; >0.60–≤0.70 poor; >0.70–≤0.80 acceptable; >0.80–0.90 excellent; >0.90 outstanding). All analyses were performed using Stata version 14.1 (StataCorp, College Station, TX).

## 3. Results

Results are presented in 3 parts addressing demographic and socioeconomic differences in diagnosis and treatment (Aim a), population-level risk of long-term CA-analgesic use (Aim b), and its distribution within patient groups by demographic, socioeconomic, and treatment characteristics (Aim c).

### 3.1. Aim (a): demographic and socioeconomic differences in diagnosis and treatment

Table 1 summarizes the demographic and socioeconomic distribution of diagnostic and treatment groups. Neuromas were rare compared with nerve injuries: among 5,625,751 individuals, 608 had a nonsurgically treated neuroma and 189 a surgically treated neuroma, compared with 3,719 nonsurgically treated and 2,122 surgically treated nerve injuries; i.e., surgically treated neuromas constituted the smallest group. Women were overrepresented among surgically treated neuromas (60%), whereas men predominated in all other groups, particularly among surgically treated nerve injuries (71%). Age distributions were broadly similar across groups, although surgically treated nerve injuries were more common at younger ages (25–34 years), while surgically treated neuromas were relatively more frequent in middle age (45–54 years). Further patient characteristics were not available in the registers.

#### 3.1.1. Demographic and socioeconomic factors—relative risks

Table 2 shows multinomial regression results for demographic and socioeconomic factors associated with diagnosis and treatment category. Women had a higher likelihood of surgically treated neuroma (RR 1.51, 95% CI 1.13–2.03) but a substantially lower likelihood of nerve injury, both nonsurgically (RR 0.64, 95% CI 0.60–0.68) and surgically treated (RR 0.42, 95% CI 0.38–0.46) nerve injuries. In addition, they also had a lower risk for nonsurgically treated neuroma (RR 0.76, 95% CI 0.64–0.89). Age did not show any association with neuroma. Younger age was associated with a slightly higher likelihood of a nonsurgically treated nerve injury, whereas older age was associated with a lower likelihood of surgically treated nerve injury. Income level showed little association with neuroma diagnoses, but nonsurgically and surgically treated nerve injuries were more common among individuals with middle income than high income. Swedish-born individuals and those living alone had a higher likelihood of a nonsurgically treated nerve injury. Swedish-born individuals had a lower likelihood of nonsurgically treated neuroma. Overall, occurrence and management of upper limb neuroma and nerve injury varied systematically by sex, age, and socioeconomic status.

### 3.2. Aim (b): long-term use of centrally acting–analgesics in the population

In the general population without neuroma or nerve injury (n = 5,619,113), 29,304 individuals (0.52%) were long-term users of CA-analgesics. The proportions were higher among patients with nonsurgically (2.14%) and surgically treated (2.12%) neuromas and among those with nonsurgically (1.77%) and surgically treated (2.73%) nerve injuries (Table 1).

Table 3 presents relative risks of long-term CA-analgesic use among individuals with neuroma or nerve injury compared with the general population. All diagnostic–treatment groups showed substantially elevated risks, approximately 3- to 5-fold (unadjusted) higher than the general population, and these excess risks remained largely unchanged after adjustment for demographic and socioeconomic factors.

The highest risk was observed among individuals with surgically treated nerve injuries (adjusted RR 6.01, 95% CI 4.63–7.81) and was lower among those with nonsurgically treated nerve injuries (adjusted RR 3.52, 95% CI 2.76–4.49). Risks were also markedly increased among patients with neuroma, both nonsurgically treated (adjusted RR 4.24, 95% CI 2.45–7.35) and surgically treated (adjusted RR 3.89, 95% CI 1.44–10.50), although estimates for neuroma were less precise due to smaller numbers. Overall, neuroma and nerve injury diagnoses were associated with a pronounced and persistent excess risk of long-term CA-analgesic use at the population level, largely independent of demographic and socioeconomic composition.

About two-thirds of the prescriptions were opioids (Table 4): tramadol/tapentadol 57%; oxycodone 22%; morphine 10%. Among the gabapentinoids (13%), pregabalin (63%) dominated. Finally, 20% of the prescriptions involved the 3 nationally recommended drugs for treating neuropathic pain (amitriptyline [50%] preferred choice [Table 4]).

### 3.3. Aim (c): differences in long-term centrally acting–analgesic use among patients

Table 5 presents ARs and ARDs for long-term CA-analgesic use among individuals with neuroma or nerve injury, stratified by demographic, socioeconomic, and treatment-related factors. Long-term CA-analgesic use occurred in fewer than 1% of the general population (approximately 0.5%) but was substantially more common among individuals, with marked variation across strata. Among surgically treated neuroma individuals, low-income individuals had ARs exceeding 10%, corresponding to an excess of nearly 10 percentage points compared with the general population; ARs of approximately 5% were also observed among both Swedish-born and immigrant individuals with surgically treated neuroma. In addition, middle-aged individuals with a nonsurgically treated neuroma presented with an AR of almost 6%. Among patients with nerve injuries, absolute risks were generally lower but remained elevated, with ARs around 3% to 4% among women, individuals aged 25 to 34 years, and those living alone with surgically treated nerve injuries, corresponding to excess risks of approximately 3 to 4 percentage points.

**Table 5:** Socioeconomic stratified absolute risk and absolute risk difference, with 95% confidence intervals, of centrally acting–analgesics (CA-analgesics; i.e., opioids, gabapentinoid drugs, amitriptyline, duloxetine, and venlafaxine used to treat neuropathic pain) of a population with a diagnosis of nonsurgically or surgically treated neuroma in upper limb as well as those with a nonsurgically or surgically treated nerve injury in the population aged 25 to 80 years, residing in Sweden by December 31, 2010 (n = 5,625,751 individuals).

Presence of nonsurgically and surgically treated neuroma or nerve injury in the upper limb															
	No neuroma or nerve injury (no nerve surgery)			Nonsurgically treated neuroma			Surgically treated neuroma			Nonsurgically treated nerve injuries			Surgically treated nerve injuries		
	N	AR	ARD	N	AR	ARD (95% CI)	N	AR	ARD (95% CI)	N	AR	ARD (95% CI)	N	AR	ARD (95% CI)
Income															
Low	1,304,696	0.73	Ref.	181	3.31	2.58 (−0.03 to 5.19)	29	10.34	9.61 (−1.47 to 20.70)	10,728	1.99	1.25 (0.99 to 1.52)	794	1.13	0.40 (−0.34 to 1.14)
Middle–high	4,357,715	0.46	Ref.	547	2.01	1.55 (0.37 to 2.73)	68	2.94	2.48 (−1.54 to 6.50)	32,690	1.20	0.74 (0.62 to 0.86)	2,136	1.12	0.66 (0.22 to 1.11)
Sex															
Men	2,823,369	0.42	Ref.	346	2.02	1.61 (0.12 to 3.09)	75	2.67	2.25 (−1.40 to 5.90)	2,299	1.26	0.84 (0.39 to 1.30)	1,516	2.24	1.82 (2.08 to 2.57)
Women	2,795,744	0.63	Ref.	262	2.29	1.66 (−0.15 to 3.48)	114	1.75	1.13 (−1.28 to 3.54)	1,420	2.61	1.98 (1.15 to 2.81)	606	3.96	3.33 (1.78 to 4.89)
COB															
Swedish-born	4,856,582	0.52	Ref.	602	1.99	1.48 (0.36 to 2.59)	78	5.13	4.61 (−0.28 to 9.51)	37,776	1.35	0.83 (0.72 to 0.95)	2,518	1.07	0.56 (0.15 to 0.96)
Immigrant	805,829	0.57	Ref.	126	3.97	3.40 (−0.01 to 6.81)	19	5.27	4.70 (−5.34 to 14.75)	5,642	1.70	1.14 (0.80 to 1.47)	412	1.46	0.89 (−0.27 to 2.05)
Age (y)															
25–34	1,002,824	0.39	Ref.	99	3.03	2.64 (−0.74 to 6.02)	34	—	—	681	1.47	1.08 (0.17 to 1.98)	526	4.18	3.79 (2.08 to 5.50)
35–44	1,167,562	0.49	Ref.	136	5.88	5.39 (1.44 to 9.35)	40	2.50	2.01 (−2.83 to 6.85)	823	1.94	1.46 (0.51 to 2.40)	548	2.37	1.88 (0.61 to 3.16)
45–54	1,131,599	0.57	Ref.	124	1.61	1.05 (−1.17 to 3.26)	52	1.92	1.36 (−2.37 to 5.09)	814	2.21	1.65 (0.64 to 2.66)	475	3.37	2.80 (1.18 to 4.43)
55–80	2,317,128	0.57	Ref.	249	—	—	63	3.17	2.60 (−1.73 to 0.93)	1,401	1.57	1.00 (0.35 to 1.65)	573	1.22	0.65 (−0.25 to 1.55)
Cohabiting status															
Living alone	2,274,412	0.64	Ref.	243	2.47	1.83 (−0.12 to 3.78)	75	1.33	0.69 (−1.90 to 3.29)	1,701	2.18	1.54 (0.84 to 2.23)	902	3.66	3.02 (1.79 to 4.24)
Cohabiting	3,344,698	0.44	Ref.	365	1.92	1.48 (0.07 to 2.88)	114	2.63	2.19 (−0.75 to 5.13)	2,018	1.44	1.00 (0.48 to 1.52)	1,220	2.05	1.61 (0.81 to 2.40)

AR, absolute risk; ARD, absolute risk difference; CI, confidence interval; COB, Country of birth.

Across most demographic and socioeconomic strata, ARs for surgically and nonsurgically treated individuals were similar or overlapping, and no consistent reduction in long-term CA-analgesic use was observed with surgical treatment. Confidence intervals were wide in neuroma subgroups, reflecting limited numbers; therefore, comparisons by treatment status are interpreted descriptively. Overall, long-term CA-analgesic use among individuals was unevenly distributed across demographic and socioeconomic groups and varied modestly by treatment context. A sensitivity analysis of RRs for use of centrally acting analgesics using the same data as in Table 4 revealed patterns of RRs like those in Table 2 for diagnosis, sex, and country of birth (Supplementary Table 2, https://links.lww.com/PR9/A447). Relative risks were generally slightly lower in the age- and income-stratified analyses for use of centrally acting analgesics.

## 4. Discussion

The nationwide register-based study shows that occurrence and management of upper limb neuromas and nerve injuries as well as long-term use of centrally acting analgesics (CA-analgesics) are socially patterned. We observed clear demographic and socioeconomic differences in likelihood of being diagnosed with nonsurgically and surgically treated upper limb neuromas or nerve injuries together with a pronounced excess risk of long-term CA-analgesic use compared with the general Swedish population. Clinical experience shows that patients with various neuromas may have different clinical profiles, like anatomic localisation, pain intensity, symptom duration, and functional impairments. This may require surgery to varying extent and with various surgical indications worldwide that may differentially influence the risk of using CA-analgesics. In our study, several surgical procedures, particularly for neuroma patients, were clustered together because studies indicate limited differences between procedures.^9,10,35^ By distinguishing diagnosis and treatment context, particularly highlighting 3 defined types of upper limb neuromas, we show that long-term pharmacological pain treatment with opioids, gabapentinoids, or substances against neuropathic pain according to Swedish guidelines is common across clinical subgroups and not confined to a single diagnostic or treatment pathway.

Limb trauma may result in nerve injuries (1%–3%), particularly impairing upper limb function.^30,38,41^ Nerve injuries were more frequent among men and younger individuals, particularly in surgically treated cases.^24,34,54^ Few studies examine demographics and socioeconomic status among individuals with a neuroma because of the lack of neuroma definition and specific-accurate ICD codes^17^ (here defined as sequelae after nerve injury and neuroma after amputations, as well as included among surgically treated neuroma^10^ those that are defined as scarred/tethered nerves^17^; latter representing an heterogenous population). Women are at higher risk for having a surgically treated neuroma probably due to our definition, which includes tethered–scarred nerves that require exploration or decompression. Age patterns differed by diagnosis, showing that surgically treated nerve injuries were less frequent at older ages, whereas no clear age gradient was observed for neuromas in contrast with earlier reports.^30,48^ Income level, country of birth, and cohabitation status also influenced diagnostic and treatment patterns, particularly for nerve injuries, highlighting the importance of accounting for population composition when assessing medication-related outcomes.

All patient groups showed markedly increased risks of long-term CA-analgesic use, reinforcing evidence that neuropathic pain after nerve injuries of various severity is common and difficult to manage.^32^ Our findings align with reports of prolonged prescribing after trauma, orthopedic surgery, and nerve entrapment procedures.^12^ The limited long-term effectiveness and dependence risks of the frequently dispensed tramadol and tapentadol remain concerns.^33,42,44,47^ Declines in tramadol use in Sweden may partly explain shifts toward stronger opioids,^3,6^ but the overall burden of long-term pharmacological treatment persists.

Neuroma surgery may reduce prolonged opioid use and surgery is recommended.^18,25,40,43,49,53^ Our observational register study showed roughly similar excess risks of long-term CA-analgesic use among nonsurgically and surgically treated neuromas. These findings should be interpreted cautiously, given the number of individuals with nonsurgically and surgically treated neuromas along with the wider confidence intervals for surgically treated neuromas. We applied an observational study design using national register data, rather than a randomized clinical study approach, which should be considered in the concluding remarks regarding neuroma surgery. One cannot exclude that early^39^ and later^9,10^ surgery can manage different pain modalities and other neuroma-related symptoms in neuroma patients to a certain extent but outcome of surgery is probably not permanent.^9,10^ Despite this, our register data indicate an associated risk of sustained pharmacological pain treatment among neuroma patients. Multimodal management strategies for neuropathic pain, including hand therapist-supervised rehabilitation, psychological support, and careful prescribing practices, is better with different opioids as a third-line treatment.^28^ Previous opioid exposure likely contributes to prolonged use, whereas surgery may reduce overuse in some patients. Individuals with preoperative use often continue treatment for longer durations.^26^

Presenting absolute risks alongside relative measures clarified both baseline and excess risks. Long-term CA-analgesic use occurred in fewer than 1% of the general population but exceeded 10% in some patient subgroups, particularly low-income individuals with surgically treated neuromas. Low income, living alone, female sex, and younger age were consistently associated with higher absolute risks. These patterns mirror broader evidence linking socioeconomic disadvantage to chronic pain, poorer health outcomes, and reduced access to nonpharmacological care.^45,46^ Living alone may reflect limited social support, whereas sex differences may relate to pain perception, health care–seeking behavior, and prescribing practices. Age-related patterns likely reflect both biological factors and treatment-selection mechanisms.

### 4.1. Methodological considerations

The outcome captured incident long-term CA-analgesic use with a defined medication coverage during 1 year and a 60-day washout period after surgery to exclude perioperative prescribing. Consequently, reported proportions underestimate overall prescribing prevalence but provide a focused measure of sustained use among individuals without prior long-term treatment. Strengths include high-quality national registers, near-complete follow-up, and estimation of both relative and absolute risks.

Limitations should be acknowledged. Neuroma definitions relied on ICD and procedure codes (see Methods) and may not capture all clinically relevant cases. The number of neuroma patients, particularly surgically treated cases, was limited, resulting in wide confidence intervals. Surgery was not stratified into various procedures, since previous data indicate that type of surgery has limited impact on outcome.^9,10,35^ Whether newly introduced surgical methods may improve outcome need to be determined.^31^ Prescription data reflect dispensed rather than consumed medications. Information on comorbidities, like psychiatric disorders, chronic pain syndromes, or cancer, was unavailable and may confound associations.^13,15,29,39^ Although prevalent long-term users were excluded, residual confounding related to baseline pain severity and symptom duration cannot be eliminated, and causal inference regarding surgical effects remains limited.

### 4.2. Clinical and public health implications

Patients with upper limb neuromas and nerve injuries face a substantial risk of persistent pain and long-term CA-analgesic use, underscoring the need for early identification and comprehensive pain management. Management and prescribing practices may differ in other health care systems, which should be investigated for generalizability of the present findings. The pronounced social gradient, especially among individuals with low income and limited social support, highlights the importance of improving access to nonpharmacological treatments and rehabilitation. Surgery alone is unlikely to prevent prolonged medication use and should be embedded within multidisciplinary care pathways. Prescribing guidelines should explicitly address socioeconomic inequalities to reduce harmful long-term medication patterns.^1,4,20,23^

## 5. Conclusion

Upper limb neuromas and nerve injuries were unevenly distributed across demographic and socioeconomic groups, but detailed patient characteristics like pain intensity and symptom duration were unknown. These nerve disorders were associated with a threefold to sixfold increased risk of long-term CA-analgesic use compared with the general population. Absolute risks revealed a substantial burden among socioeconomically disadvantaged individuals, underscoring the need for equitable, multimodal strategies to prevent and manage persistent neuropathic pain after upper limb nerve injuries.

## Disclosures

The authors have no conflict of interest to declare.

## Supplemental digital content

Supplemental digital content associated with this article can be found online at https://links.lww.com/PR9/A447.
