# Supplementary material for: Long-term analgesic use after neuroma and upper limb nerve injury: patterns in Sweden
Source: Pain Rep. 2026 Oct 28;11(6):e1511. doi: 10.1097/PR9.0000000000001511 (PMC13719113; doi:10.1097/PR9.0000000000001511)
Supplement: Supplementary file 1 [file painreports-11-e1511-s001.pdf]

Supplemental Table 1. Definition of nerve injuries and neuroma (ICD-10) and surgical procedures (Swedish version of the NOMESCO Classification of Surgical Procedures; NCSP) used in the present study.

Nerve injuries – non-surgically treated:

ICD-codes

S.44	Injury of nerves at shoulder and upper arm level
S.54	Injury of nerves at forearm level
S.64	Injury of nerves at wrist and hand level

Nerve injuries – surgically treated:

ICD-codes

S.44	Injury of nerves at shoulder and upper arm level
S.54	Injury of nerves at forearm level
S.64	Injury of nerves at wrist and hand level

Surgical codes

ACB21	Suture of peripheral nerve – median nerve
ACB22	Suture of peripheral nerve – radial nerve
ACB23	Suture of peripheral nerve – ulnar nerve
ACB29	Suture of peripheral nerve – other or unspecified nerve.
ACC21	Reconstruction of peripheral nerve – median nerve
ACC22	Reconstruction of peripheral nerve – radial nerve
ACC23	Reconstruction of peripheral nerve – ulnar nerve
ACC29	Reconstruction of peripheral nerve - other or unspecified nerve.

Neuroma – non-surgically treated:

ICD-codes

T92.4	Sequelae of injury of nerve of upper limb
T87.3B	Neuroma of amputation stump shoulder/arm
T87.3C	Neuroma of amputation stump elbow/forearm
T87.3D	Neuroma of amputation stump wrist/hand

Neuroma – surgically treated:

ICD-codes

T92.4	Sequelae of injury of nerve of upper limb
T87.3B	Neuroma of amputation stump shoulder/arm
T87.3C	Neuroma of amputation stump elbow/forearm
T87.3D	Neuroma of amputation stump wrist/hand

Surgical codes

ACB21	Suture of peripheral nerve – median nerve
ACB22	Suture of peripheral nerve – radial nerve
ACB23	Suture of peripheral nerve – ulnar nerve
ACB29	Suture of peripheral nerve – other or unspecified nerve.
ACC21	Reconstruction of peripheral nerve – median nerve
ACC22	Reconstruction of peripheral nerve – radial nerve
ACC23	Reconstruction of peripheral nerve – ulnar nerve
ACC29	Reconstruction of peripheral nerve - other or unspecified nerve

ACA19 Exploration of peripheral nerve - other or unspecified nerve
ACB19 Excision of lesion of peripheral nerve - other or unspecified nerve
ACC19 Transection of peripheral nerve - other or unspecified nerve
ACC49 Transposition of peripheral nerve - other unspecified nerve
ACC59 Decompression and freeing of adhesions of peripheral nerve - other or unspecified nerve
ACC99 Other operation for impaired function of peripheral nerve - other or unspecified nerve
ACA13 Exploration of peripheral nerve - ulnar nerve
ACB13 Excision of lesion of peripheral nerve – ulnar nerve
ACC13 Transection of peripheral nerve – ulnar nerve
ACC43 Transposition of peripheral nerve - ulnar nerve
ACC53 Decompression and freeing of adhesions of peripheral nerve - ulnar nerve
ACC93 Other operation for impaired function of peripheral nerve – ulnar nerve
ACA11 Exploration of peripheral nerve – median nerve
ACB11 Excision of lesion of peripheral nerve – median nerve
ACC11 Transection of peripheral nerve – median nerve
ACC41 Transposition of peripheral nerve – median nerve
ACC51 Decompression and freeing of adhesions of peripheral nerve – median nerve
ACC91 Other operation for impaired function of peripheral nerve – median nerve
ACA12 Exploration of peripheral nerve – radial nerve
ACB12 Excision of lesion of peripheral nerve – radial nerve
ACC12 Transection of peripheral nerve – radial nerve
ACC42 Transposition of peripheral nerve – radial nerve
ACC52 Decompression and freeing of adhesions of peripheral nerve – radial nerve
ACC92 Other operation for impaired function of peripheral nerve – radial nerve

Supplemental Table 2. Sensitivity analysis of the relative risks of use of centrally acting analgesics (CA-analgesics; i.e., opioids, gabapentinoid drugs and the three drugs amitriptyline, duloxetine and venlafaxine used to treat neuropathic pain) in non-surgically or surgically treated neuroma or nerve injury in upper limb in a Swedish population (25 - 80 years, residing in Sweden by December 31st, 2010) based on data used for the evaluation of Absolute Risk (AR) and Absolute Risk Difference (ARD) (Table 5). The relative risk (RR) and 95% confidence intervals (CI) are presented as unadjusted (Model 1) and adjusted (Model 2) values.

		Model 1 (unadjusted)	Model 2 (adjusted)
**Cases**	***Controls***	Reference	Reference
	Non-surgically treated neuroma	4.17 (2.41-7.22)	4.25 (2.45-7.37)
	Surgically treated neuroma	4.12 (1.53-11.11)	3.88 (1.44-10.470)
	Non-surgically treated nerve injury	3.45 (2.70-4.40)	3.59 (2.81-4.58)
	Surgically treated nerve injury	5.36 (4.13-6.96)	6.03 (4.64-7.83)
**Sex**	*Men*		Reference
	Women		1.46 (1.43-1.49)
**Age (years)**	*25-34*		Reference
	35-44		1.27 (1.22-1.33)
	45-54		1.45 (1.40-1.51)
	65-80		1.57 (1.52-1.63)
**Income**	*Low*		1.63 (1.59-1.68)
	Middle-high		Reference
**Country of birth (COB)**	*Immigrant*		Reference
	Swedish-born		1.06 (1.02-1.09)
